# The iranian model as a potential solution for the current kidney shortage crisis

**DOI:** 10.1590/S1677-5538.IBJU.2018.0441

**Published:** 2019

**Authors:** Bahar Bastani

**Affiliations:** 1 Division of Nephrology, Saint Louis University Health Science Center, Saint Louis, MO, USA

*To the editor,*

Kidney transplantation is the treatment of choice for eligible patients who have reached end stage renal disease (ESRD). It significantly improves patient survival and quality of life, and reduces cost of health care when compared to dialysis. Six decades of success in the field of transplantation have made it possible to save thousands of lives every year. However, we have been facing an ever - growing shortage of organs in the recent years. The supply of kidneys available for transplantation has not come close to an ever - increasing demand for transplantation. In the United States there are currently more than 100.000 patients waiting for kidney or kidney - pancreas. However, the supply of kidneys that stagnated around 16.000 to 17.000 per year (10.000 - 11.000 deceased donor and around 6.000 live donor kidneys per year) in the decade between 2004 and 2014 has since then increased to near 20.000 kidneys in 2017, predominantly secondary to increased availability of deceased donor kidneys from drug overdose deaths (opioids addiction crisis) in the USA (1, 2). There has been a decline in the annual number of living donor kidneys from a peak of 6.648 in 2004 to only 5.813 in 2017. Moreover, while only 20% of the half million dialysis patients in USA make it to transplant wait list, of them 5.000 die each year waiting (an annual mortality rate of around 7% among patients waiting for a kidney) (1). The ever - widening gap between demand and supply has resulted in an illegal black market and unethical transplant tourism of global proportions (3, 4).

The global organ crisis has led to transplant tourism and a black market in kidneys that creates dangerously high risks for all involved. Rich patients from the United States, Canada, Europe, Japan, Australia, Israel, Saudi Arabia, and Oman travel to countries like China, the Philippines, Pakistan, India, Brazil, Bolivia, Iraq, Moldova, Peru, Turkey, and Colombia, and pay black market brokers tens to hundreds of thousands of dollars to buy kidneys (3, 4). These kidneys are obtained from executed prisoners (around 90% of kidneys in China) or illegally obtained from desperately poor locals, with no guarantee that they will be informed of the medical or legal risks they are taking. The transplants are often performed under unsafe conditions without proper donor and recipient evaluation. Under such circumstances, recipients are at risk of getting suboptimal organs, transferable diseases (Hepatitis B, Hepatitis C, HIV) and run a high risk that organ traffickers will cheat them. There is even a chance that they will be prosecuted for illegal activity once they return to their home countries. Donors run the highest risks, they go without adequate post - operative care, are frequently cheated by organ brokers, and lack any form of legal support if cheated or mistreated. Moreover, they run the risk of being arrested for having participated in an illegal activity.

The Iranian Model of regulated incentivized live kidney donation has evolved over the past 35 years and provides an example of a nation willing to take a novel approach to solving its kidney shortage (5-7). The result of the Iranian Model has not always been positive but, to its credit, the Iranian medical community has responded by continually adjusting the system to deal with problems as they arose - banning organ sales to foreigners, demanding more government benefits for both donors and recipients, and providing ever more comprehensive guidelines for the donor vetting process (8, 9). Health authorities in Iran should continue to improve the system by alleviating the shortcomings of their program and through more vigilant enforcement of the existing laws, regulations, and guidelines. The system is clearly in need of better pre - donation evaluation and long - term follow-up of donors, as well as more social support networks for donors, life - long health insurance, and more financial compensation for donors. The Iranian Model has achieved several important milestones that other nations should learn from:

1. Created a legal structure for enforceable donor / recipient contracts.
2. Provided guidelines to help ensure informed consent for donors and their next of kin.
3. Licensed NGOs (Anjomans) to provide free assistance to both recipients and donors in brokering transplant deals and applying for related government and charitable benefits.
4. The requirement of the same citizenship between donors and recipients to prevent transplant - tourism and protect Iran's own ESRD patients from being excluded in favor of potentially higher paying foreigners.
5. Initiated important data collection and studies to evaluate its system of paid donation.
6. Funded through the government, all transplant related medical care cost is covered.
7. Requirement that transplantations be performed at university hospitals and by qualified transplant teams.

As a result of all these achievements, unlike anywhere else in the world, everyone who medically qualifies for a transplant in Iran can begin the process for getting one, and in some regions of the country there is even a waiting list for people who want to donate. While there is much room to improve the Iranian Model of regulated incentivized live kidney donation, with some significant revisions, the Iranian Model could serve as an example for how other countries could make significant strides to lessening their own organ shortage crises.

An important objection to the Iranian Model has been that it allows coercion of the poor because of their poverty by the rich. The question to keep in mind is what such potential donors will do if deprived of the opportunity to donate in exchange for a reasonable financial incentive. What could they do instead to alleviate their financial predicament, prevent impending homelessness, help a sick relative, or avoid imprisonment for failure to pay a tort judgment? What other courses of action are available and at what cost to themselves and to the society? If the alternatives were incarceration, loss of a loved one, stealing, murder, drug trafficking, suicide, or homelessness, then donating a kidney in exchange for a fair financial incentive and saving a life would be a more reasonable and honorable choice, even if the relief is partial or temporary.

Another important objection to a legal system of regulated government incentivized living unrelated kidney donation is that it will result in disappearance of altruistic living related and deceased donor donations. However, the experience in Iran has shown that with the improvement in the required infrastructure the percentage of deceased kidney transplantations has increased over time (10, 11). Moreover, availability of a living unrelated kidney transplantation reduces the potential emotional coercion and ill family dynamics, as well as, potentially jeopardizing a family member who may have a similar genetic or environmental predisposition to chronic kidney disease.

In order to alleviate some of the short comings of the Iranian Model, the direct financial relationship between the potential donor and recipient should be avoided by either changing the current scheme to a fully government compensated system, or alternatively by creating an organ bank run by state health authorities (Kidney foundation). Paid donors should not be allowed to choose their recipient. This decision should be completely at the discretion of the health authorities, the transplant team, and based on the health condition of the recipients and compatibility of the donors (through a similar scoring / priority system as the one used by United Network for Organ Sharing (UNOS)). No alternatives or independent method for obtaining organs other than altruistic donations from family members should be allowed. Once such modifications are implemented there is a chance that the Iranian model could become an example for other countries, but not before. Moreover, the donors should receive a life - time health insurance, and a comprehensive long - term follow-up. While the immediate cash provided often seems to alleviate urgent financial needs, more efforts are needed to help ensure long-term benefits.

In an electronically published (ahead of print, in June 20, 2018) article by Yacoubian, et al. in this journal, the authors review the possibility of adopting financially derived live donor kidney transplantation (12). After reviewing all different alternatives they conclude that it is time to revisit the idea of governmental supervision with a regulated compensation to living donors, especially that no alternative solution has been available up to now to alleviate the current ever worsening organ shortage crisis (12).
